# E-cadherin Beyond Structure: A Signaling Hub in Colon Homeostasis and Disease

**DOI:** 10.3390/ijms20112756

**Published:** 2019-06-05

**Authors:** Amanda C. Daulagala, Mary Catherine Bridges, Antonis Kourtidis

**Affiliations:** Department of Regenerative Medicine and Cell Biology, Medical University of South Carolina, 173 Ashley Avenue, Charleston, SC 29425, USA; gunarath@musc.edu (A.C.D.); bridgmar@musc.edu (M.C.B.)

**Keywords:** adherens junctions, CDH1, cadherin, catenin, colorectal cancer, inflammatory bowel disease, epithelial, colon crypt, microbiome

## Abstract

E-cadherin is the core component of epithelial adherens junctions, essential for tissue development, differentiation, and maintenance. It is also fundamental for tissue barrier formation, a critical function of epithelial tissues. The colon or large intestine is lined by an epithelial monolayer that encompasses an E-cadherin-dependent barrier, critical for the homeostasis of the organ. Compromised barriers of the colonic epithelium lead to inflammation, fibrosis, and are commonly observed in colorectal cancer. In addition to its architectural role, E-cadherin is also considered a tumor suppressor in the colon, primarily a result of its opposing function to Wnt signaling, the predominant driver of colon tumorigenesis. Beyond these well-established traditional roles, several studies have portrayed an evolving role of E-cadherin as a signaling epicenter that regulates cell behavior in response to intra- and extra-cellular cues. Intriguingly, these recent findings also reveal tumor-promoting functions of E-cadherin in colon tumorigenesis and new interacting partners, opening future avenues of investigation. In this Review, we focus on these emerging aspects of E-cadherin signaling, and we discuss their implications in colon biology and disease.

## 1. Introduction

### 1.1. The Adherens Junctions

Cell-cell adhesion complexes are indispensable for tissue integrity and organ function; however, their disruption can lead to numerous diseases, including inflammation and cancer. The Adherens Junction (AJ) is a major cell-cell adhesion structure, key for maintaining tissue integrity and architecture through its intimate tethering to the actin and microtubule cytoskeleton [1]. The core components of AJs are the members of the classical cadherin superfamily, such as epithelial cadherin (E-cadherin), neural cadherin (N-cadherin), placental cadherin (P-cadherin), as well as members of the catenin family of proteins, namely p120 catenin (p120), α-catenin, and β-catenin [2]. Nectin is another important cell-cell adhesion molecule present at the AJs, which binds intracellularly to Afadin via its C-terminus [3].

The cadherin superfamily includes classical, desmosomal, protocadherins, and unconventional types of cadherins [2,4,5]. In this review, we focus on the classical type I cadherin E-cadherin, which is the predominant member of the family in epithelial tissues and is encoded by the *CDH1* gene. Classical mammalian cadherins have five extracellular domains, spanning EC1 to EC5, with calcium-binding sites. Each of these sites contain negatively charged motifs that can bind to three Ca^2+^ molecules, thus strengthening the interactions between the extracellular domains [4,6]. The homophilic binding of EC1 domains between cells is known as “trans” interactions; binding of the EC1 domain of one cadherin molecule to the EC2 domain of another within the same cell is known as “cis” interactions. Both cis and trans interactions are important for the formation of cadherin-based adhesions [6].

Armadillo repeats are homologous tandem repeats of approximately 40 amino acids, a defining characteristic of β-catenin and p120. The cytoplasmic carboxy terminal region of E-cadherin binds with β-catenin, which, in turn, interacts with α-catenin [7]. The “PEST” sequence of type I cadherins is subjected to rapid turnover via the action of ubiquitin ligases. However, this motif overlaps with the β-catenin binding region, thus preventing cadherins from proteasomal degradation when bound to β-catenin [7]. α-catenin binds to the 118–149 amino acid sequence of β-catenin. Further, it binds to F-actin via its 697–906 amino acid sequence and to Afadin, another actin-associated protein, through its 391–631 amino acid sequence in the M-domain [7,8]. In addition, α-catenin has a homologous region to another actin-binding protein known as Vinculin [4]. p120 is also involved in cytoskeletal dynamics through interaction with small GTPases [9]. Importantly, p120 is essential for the stability of cadherin junctions. p120 binds to the juxtamembrane domain (JMD) of E-cadherin, which blocks binding of the ubiquitin ligase Hakai, protecting E-cadherin from endocytosis and turnover [10,11,12]. p120 downregulation causes downregulation of E-cadherin and negatively affects morphology of SW48 colorectal adenocarcinoma epithelial cells [13]. Restoration of p120 significantly enhances epithelial morphology and E-cadherin levels [13]. A more recently identified protein named PLEKHA7 (Pleckstrin Homology domain-containing, family A member 7) binds to the N-terminus of p120 at the AJs and to the minus ends of microtubules through a protein termed Nezha [14]. PLEKHA7 is also critical in stabilizing the actin cytoskeleton and the overall integrity of the AJs, potentially through interaction with several cytoskeletal components at the AJs, such as Actin, α-actinin (ACTN1), and myosin light chain 6 (MYL6) [15,16].

Although cadherin-based junctions form across lateral areas of cell-cell contact, mature adherens junctions are found at the apical areas of cell-cell contact in polarized differentiated epithelial cells and tissues, where they also tether to an apical circumferential actin ring, forming a structure called the zonula adherens (ZA) [1]. The ZA is in close proximity and closely related to the tight junctions (TJ), the cell-cell adhesion complex that is primarily responsible for the barrier function of epithelial tissues [17]. For example, several components of the ZA, such as PLEKHA7, associate with TJ components such as ZO-1 and Cingulin, affecting barrier function [16,17]. In addition, the ZA and the TJs are tethered through the actin circumferential ring [18,19]. Importantly, E-cadherin is required for TJs and tissue barrier formation [20,21]. Therefore, E-cadherin is a quintessential molecule for enabling of the core function of epithelial tissues, which is formation of a tissue barrier. This is well understood in the context of intestinal tissues, such as the colon.

### 1.2. The Colonic Crypt 

The colon, or large intestine, is the part of the digestive system primarily responsible for the absorption of water and electrolytes that remain after nutrient absorption in the small intestine, and to passage stool. Anatomically, the colon continues from the small intestine to the segment called the cecum, which is followed by the ascending colon, the transverse colon, the descending colon, the sigmoid colon, and the rectum. The colonic wall is covered by a columnar epithelial monolayer called the mucosa, which contains invaginations called crypts. The epithelial monolayer is supported by a basement membrane and an underlying layer of connective tissue called lamina propria. The existence of crypts is also a feature of the small intestine; however, colonic crypts do not extend into villi structures, which specifically appear in the small intestinal tissue. The colonic crypt is a well-organized and intriguing structure that contains a gradient of distinct subpopulations of different cell types: an Lgr5+ stem cell niche that lies at the base of the crypt and produces adjacent progenitor cells, which, in turn, progressively fully differentiate towards the apical part of the crypt to colonocytes (or absorptive cells), to the mucus-secreting goblet cells, to the peptide hormone-secreting endocrine cells, and to the Paneth cells that are occasionally found in the ascending colon [22]. This structure provides the colon with a robust renewal mechanism: the intestinal epithelium has a turnover rate of four to five days, making it the tissue with the fastest turnover in the human body. This mechanism allows the colon to maintain homeostasis under the harsh conditions of the intestinal lumen, which induces constant cell shedding from the top of the crypt [23]. Accordingly, this cell gradient across the crypt is accompanied by a signaling gradient. Two major signaling pathways that determine cell fate in the colonic crypt are the Wnt and the BMP signaling pathways. The Wnt signaling is activated at the bottom and is gradually suppressed towards the top part of the crypt; in contrast, BMP signaling is activated at the top of the crypt [24,25,26]. This elegant balance allows for the maintenance of a stem cell niche at the bottom of the crypt, giving rise to cells that proliferate and eventually fully differentiate at the top of the crypt. 

E-cadherin is the main cadherin expressed in the colonic crypt epithelium. E-cadherin is vital for the proper morphogenesis of the intestine [27]. E-cadherin expression across the developed crypt is not uniform; E-cadherin levels are lower towards the base of the crypt but are strongly expressed at the apical part of the crypt, which supports the formation of the intestinal barrier, an essential function of the organ [26,28]. This E-cadherin expression gradient is consistent with the state of differentiation of the crypt cells, which occurs at the top part of the crypt as well as with the activation status of Wnt signaling. Indeed, β-catenin is nuclear and Wnt is active at the bottom of the crypt; however, Wnt signaling becomes gradually inactive towards the top of the crypt due to increased APC (Adenomatous Polyposis Coli) expression, resulting in β-catenin association with E-cadherin and cell-cell junction stabilization at the differentiated cell compartment at the top of the crypt [26,28]. Nevertheless, there are heterogeneous crypts with clusters of cells towards the bottom of the crypt that strongly express E-cadherin [28]. It is not clear why these cells express high E-cadherin and what type of cells these are. It has been suggested that high E-cadherin expression may serve in stabilizing contacts between stem cells and surrounding cells at the bottom of the crypt, and that E-cadherin expression is suppressed in the proliferating cells to allow them to progress towards the top part of the crypt [28]. Ephrin EphB - ADAM10 - mediated shedding of E-cadherin results in compartmentalization of E-cadherin contacts, which is critical in fine-tuning cell migration and proper organization of cells in the crypt [29]. Furthermore, E-cadherin stabilization promotes colony formation of colonic stem cells [30]. Interestingly, a recent work showed that E-cadherin is required for Lgr5+ gastric stem cell survival [31]. However, association of E-cadherin with the Lgr5+ colonic stem cells has yet to be established. The role of E-cadherin in stem cell survival and its potential repercussions in cancer stem cell survival and pro-tumorigenic transformation has been explored in a computational model [32]; however, this also has yet to be experimentally tested.

## 2. Colorectal Cancer and E-cadherin

### 2.1. E-cadherin is a Double-Faced Signaling Molecule in the Colon

Although the fast renewal capacity and turnover of the colonic epithelium provides plasticity and the ability to maintain homoeostasis, it also makes the colon susceptible to mutagenesis and potentially tumorigenesis. Indeed, colorectal cancer (CRC) is the third most prevalent and second deadliest form of the disease [33], and most cancers in the colon arise from the mucosal epithelial layer. Preventative screening has led to a gradual decrease in CRC incidence in recent decades, especially among older populations. However, studies conducted in cancer patients diagnosed from 1995 to 2014 in the USA showed a surprising increase in CRC incidence rates in young populations [34,35]. This and other studies have suggested a link between increased obesity rates and colon cancer. In a recent work, it was demonstrated that obesity and ensuing diabetes and hyperglycemia negatively impact intestinal barrier function, which, in turn, results in microbial infection and inflammation, a common precursor to CRC [36]. However, incidents in East Asia, where a lower obesity level has been observed in comparison with the global numbers [37], the most prevalent cancer type remains CRC, suggesting other contributing factors to the disease.

**Figure 1:**
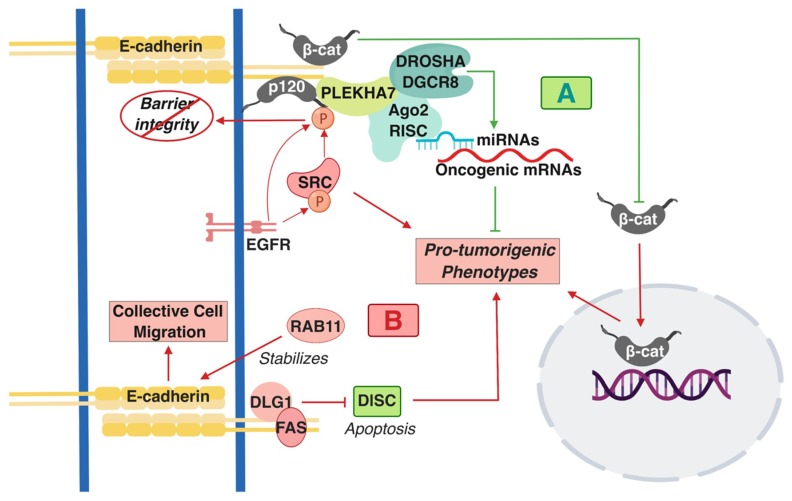
A summary of the pro- and anti-tumorigenic signaling mediated by E-cadherin-based Adherens Junctions AJ complexes that has been described in colon cells and tissues. (**A**) Shades of green/blue depict tumor-suppressing components and functions, whereas (**B**) shades of red represent the tumor-promoting ones described in the text. T-arrows represent inhibition of molecules or pro-tumorigenic signaling processes; straight arrows represent activation of molecules or pro-tumorigenic signaling processes. β-cat: β-catenin; p120: p120 catenin; RISC: RNA-induced silencing complex.

The status of E-cadherin has been extensively studied in CRC in the context of Wnt/β-catenin signaling because dysregulation of this pathway is a predominant driver of tumorigenesis in the colon [38,39,40]. Overall, E-cadherin has a predominantly tumor-suppressing role in this context. For example, E-cadherin suppresses the pro-tumorigenic transformation that is promoted by β-catenin activating mutations by keeping β-catenin at areas of cell-cell contact as opposed to allowing it to go to the nucleus (Figure 1A) [41,42]. However, other functions of E-cadherin have recently emerged with regards to its role in colon tumorigenesis. Interestingly, many of these studies point towards signaling directly driven by the AJs and not indirectly in the nucleus, through the release of β-catenin. One interaction that regulates this signaling is the cross-talk of cadherin complexes with EGFR and Src. Src is an oncogenic non-receptor tyrosine kinase that is overexpressed and/or activated in colon tumors and is one of the major drivers in colon tumorigenesis [43,44,45]. Both EGFR and Src can directly phosphorylate p120 [46,47]; this interaction and overall Src activity disrupts strong adhesion, resulting in compromised barrier function (Figure 1B) [48,49]. However, this interaction also has consequences in promoting pro-tumorigenic cell behavior. Disruption of cadherin-mediated adhesion promotes metastatic and invasive phenotypes (Figure 1B) [43,49].

E-cadherin and p120 are required for Src-dependent, anchorage-independent growth and downstream suppression of RhoA signaling [50]. p120 acts as an obligatory haploinsufficient tumor suppressor, whereby one allele of p120 is required for early stages of tumorigenesis in the intestine in *Apc*-mutated mouse models [51]. Findings also imply a similar role for E-cadherin. Another work has shown that E-cadherin forms a complex together with the polarity component DLG1 and with the cell death regulator FAS at areas of cell-cell contact (Figure 1B) [52]. This interaction suppresses apoptosis of the HCT15 colon cancer cells by inhibiting the formation of the pro-apoptotic, death-inducing signaling complex (DISC), which signifies a pro-survival role of E-cadherin in colon cancer cells (Figure 1B) [52]. E-cadherin-positive cells and tumors appear chemotherapy-resistant [53,54]. These studies challenge the dogma of Epithelial-to-Mesenchymal Transition EMT-mediated cancer progression. One study demonstrated that L1-induced metastasis of colon cancer cells is E-cadherin and EMT-independent [55], whereas a more recent work has shown that Rab11 stabilizes E-cadherin levels and promotes collective cell migration of colon cells (Figure 1B) [56,57]. These findings unravel a tumor-promoting role of E-cadherin complexes, contrary to the prevailing notion that E-cadherin is a *de facto* tumor suppressor. In attempts to reconcile these findings, it was demonstrated that there are distinct E-cadherin complexes at the AJs of polarized monolayers of the well-differentiated colon epithelial Caco2 cells: an apical-specific complex with tumor suppressing properties and a basolateral-specific that promotes pro-tumorigenic behavior, dependent on Src activity and Src-mediated p120 phosphorylation [16]. This work led to another revelation regarding cadherin-mediated signaling, demonstrating that E-cadherin-p120 complexes, though their interacting partner PLEKHA7, recruit the core and accessory components of the RNA interference (RNAi) machinery, including DROSHA, DGCR8, Ago2, and the RNA-induced silencing complex (RISC) at the apical AJs of the well-differentiated colon Caco2 cells. Cadherins can regulate miRNA processing and activity to suppress expression of a series of pro-tumorigenic factors and anchorage-independent growth (Figure 1A) [15,16,58]. In summary, the above studies have altered our perception on the role of cadherin complexes in cancer by: a) demonstrating that E-cadherin-based complexes can also act as tumor promoters; b) revealing that E-cadherin complexes are signaling hubs and not merely structural components of cells. It would be of interest to examine the extent to which these interactions occur in colon cells and tumors and how they contribute to the tumor suppressing or tumor promoting functions of E-cadherin.

### 2.2. E-cadherin as a Colon Cancer Biomarker?

E-cadherin has been proposed as an additional biomarker for CRC because of its downregulation or loss in many cancers [59]. Currently, the Carcinoembryonic Antigen (CEA) is the most commonly used CRC marker. Other markers in serum or plasma such as *APC* and *KRAS* mutations, DNA integrity, histone and DNA methylation, and some microRNAs have also been suggested as CRC biomarkers [60,61,62]. A meta-analysis reported that low or lost E-cadherin levels in CRC correlate with poor prognosis in Asian patients but not in European patients [63]. Signet ring cell carcinoma (SRCC) is a rare adenocarcinoma that primarily occurs in the stomach and occasionally in the colon [64]. The World Health Organization (WHO) defines SRCC as the cancer type where >50% of tumor cells have intracytoplasmic mucin present [65]. A study that investigated 59 patients reported a statistically significant higher survival for patients with E-cadherin positive SRCC when compared with lower survival rates of patients with E-cadherin negative SRCC [64]. In addition, increased levels of soluble plasma E-cadherin, which would indicate E-cadherin cleavage and compromised cell-cell adhesion, has been associated with advanced stage colorectal cancer and with familial adenomatous polyposis (FAP), a rare condition in the colon that strongly predisposes for CRC [66]. However, in the same study, plasma E-cadherin levels were unaltered in patients with inflammatory bowel disease (IBD) or early stage colorectal tumors [66]. Similarly, although E-cadherin loss was found to strongly predict lymph node-positive colorectal cancers [67], another study found no statistically significant correlation of reduced E-cadherin expression with development of metastatic colon disease [68] and loss of membranous expression of E-cadherin, which would indicate junction-bound cadherin was not significantly correlated to Duke’s staging, tumor grade, sex, size, and site of tumor [69]. Furthermore, E-cadherin is still expressed in several colon cancer cell lines [70]. HCT116 and HT-29 cells in 3D cultures adopt an invasive phenotype without progressing through EMT while continuing to express robust levels of E-cadherin [71]. Together, these data are in agreement with the recent conflicting findings in E-cadherin signaling in colon tumorigenesis and further challenge the traditional view of E-cadherin as a tumor suppressor. They also suggest that the use of additional markers that broadly incorporate other members of cadherin complexes that modulate E-cadherin’s barrier and signaling functions in the colon is required to provide a better association with disease initiation and progression.

### 2.3. The Role of Other Cadherins in Colon Tumorigenesis

Although absent in normal colon tissues, P-cadherin is abnormally expressed early in colorectal carcinogenesis, promoting colonic crypt fission and metastasis in the liver [72,73,74]. Similarly, N-cadherin is upregulated in a cohort of colon tumors [75]. Interestingly, this expression coincides with E-cadherin expression and is independent of expression of EMT promoters, such as SNAI1 and TWIST [75]. This, together with other studies [16] that show retention of E-cadherin and simultaneous overexpression of mesenchymal cadherins, such as N-cadherin or Cadherin-11, also demonstrates a deviation from the classical model of EMT in tumorigenesis. Heterotypic cadherin interactions may drive tumor cell migration and metastasis, as was recently shown in other epithelial cancer cell types [76]. It remains to be shown whether this is the case in the colon. Nevertheless, a role of N-cadherin in promoting colon myofibroblast migration and invasion upon TGF-β stimulation has been demonstrated [77]. Increased expression of Cadherin-11, which is another mesenchymal cadherin, has also been shown to promote pro-tumorigenic signaling in Caco2 cells [16] or cell migration in HT-29 cells [78]. Interestingly, Cadherin-11 expression is increased in patients with IBD, although the significance of this finding in cell signaling and behavior has yet to be explored [79]. Overall, the status and role of cadherins other than E-cadherin in the colon and in colon tumorigenesis is understudied in comparison with other epithelial tumors, leaving this an open field of investigation.

## 3. E-cadherin in Inflammatory Bowel Disease

**Figure 2:**
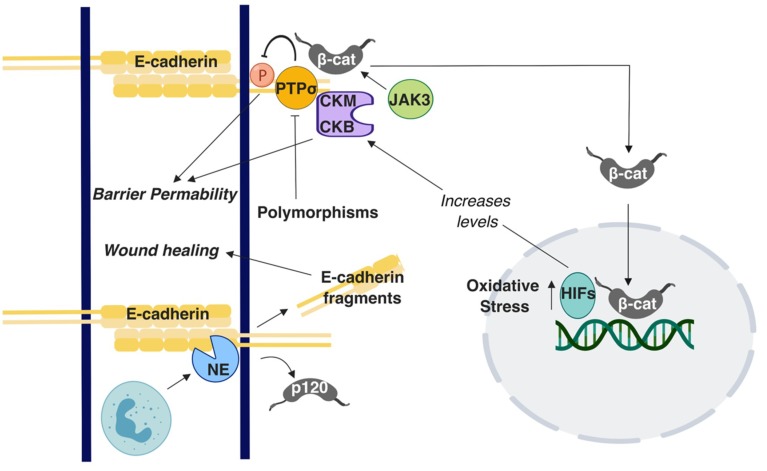
A summary of the E-cadherin-mediated signaling and interactions involved in Inflammatory Bowel Disease IBD. T-arrows represent inhibition of molecules or processes; straight arrows represent activation of molecules or processes. β-cat: β-catenin; p120: p120 catenin.

A suggested culprit for the increased incidence of colon cancer in younger ages is increased cases of Inflammatory Bowel Disease (IBD) [80], which is a general term for two conditions: Ulcerative Colitis (UC), which is predominantly found in the large intestine, and Crohn’s Disease (CD), which occurs in both the small and large intestine [81]. The reasons of increased IBD in the general population are still not well understood. However, IBD patients carry a significantly increased risk for developing CRC [82]. The intestinal barrier is compromised in IBD, allowing the flux of water and dissolved solutes, ions, and nutritional molecules across the intestinal barrier [83,84]. Because E-cadherin is key to barrier maintenance, its dysregulation could increase the risk of developing IBD and ultimately CRC. Indeed, genome-wide association studies have shown the E-cadherin gene *CDH1* as a susceptibility locus in UC [85], along with *HNF4* and *LAMB1* [86]. N-terminal truncation of E-cadherin due to polymorphisms in *CDH1* results in cytoplasmic aggregation of E-cadherin in CD while indirectly mis-localizing β-Catenin [87]. Mutations in other genes can also affect AJ- associated proteins. Polymorphisms in the *C1orf106* gene is a risk factor in UC [88]. A study conducted using *C1orf106*^−/−^ colonic organoid-derived epithelial cells noted decreased surface E-cadherin levels and increased intracellular E-cadherin levels [88]. Furthermore, *C1orf106*^−/−^ cellular monolayers exhibited increased permeability in luciferase permeability and trans-epithelial electrical resistance (TEER) assays [88]. In the same study, *C1orf106*^−/−^ mice demonstrated impaired recovery from DSS-induced colitis and damaged colon crypts when *Citrobacter rodentium* was introduced in comparison with *C1orf106*^+/+^ mice [88]. Additionally, in a Chloride channel protein-2 (CIC-2; *CLCN2*) null mouse model, recovery from DSS-induced colitis was impaired and the epithelial permeability was decreased [89]. Although these results were firstly attributed to compromised TJ function [89], it was subsequently shown that the AJs were also responsible [90]. E-cadherin and β-catenin distribution as well as the ultrastructural tissue morphology were specifically altered in the colon while it was retained in small intestine [90]. These observations suggest that CIC-2 is associated with the AJs’ function specifically in the colon [90]. Polymorphisms in the receptor-type tyrosine-protein phosphatase-S gene (*PTPRS*) that encodes for the PTPσ protein are associated with UC. Importantly, PTPσ has been demonstrated to localize at the apical region. E-cadherin and β-catenin act as substrates for PTPσ in the brain and epithelial barrier is perturbed due to tyrosine phosphorylation; accordingly, it has been suggested that polymorphisms in the *PTPRS* gene can cause disruption in the apical junctions in the colon, promoting UC [91] (Figure 2). Earlier work has shown hypermethylation in the *CDH1* promoter region and CpG island methylation of *CDH1* in UC conditions [92,93]. Although epigenetic regulations of AJ proteins, especially of E-cadherin, have been extensively studied in the context of CRC, there is overall limited knowledge available on this topic regarding IBD.

E-cadherin regulates colon homeostasis also through interactions with immune cells. CD11c+ mononuclear phagocytes in an IBD mouse model have higher than usual number of adhesions to the epithelium due to upregulated E-cadherin expression, leading to inflammation [94]. Polymorphonuclear neutrophils (PMNs) are a type of white blood cells that have been shown to affect mucosal barrier during the inflammation process by altering the localization patterns of E-cadherin and β-catenin, eventually leading to perturbation of AJs [95]. A recent study demonstrated that E-cadherin is enzymatically cleaved to several peptide fragments by neutrophil elastase (NE), a known inflammatory protease present in IBD (Figure 2). These peptide fragments were present in the patient tissues sample analyzed in the study and could enter the cytosol of Caco2 cells *in vitro* by crossing the lipid bilayer. Although these fragments did not alter proliferation rates, they improved wound healing in in vitro assays (Figure 2) [96]. Although E-cadherin fragmentation would seemingly impair barrier function and exacerbate IBD, the faster wound healing could instead be beneficial for IBD; the action of these fragments implies downstream signaling, which warrants further investigation. 

Although not extensively investigated as E-cadherin, studies have investigated the roles of other AJ proteins in IBD, demonstrating that E-cadherin, p120, and α-catenin expression is downregulated in the colonic mucosa of IBD patients [97]. In contrast, another study showed focal increases of the E-cadherin - β-catenin complex in the mucosa of IBD patients, suggesting a putative defensive response against inflammation [98]. An in vivo study reported that p120 loss caused inflammation due to increased association of neutrophils with the disturbed epithelial barrier [99]. Similarities were noted between the p120-ablated phenotype and IBD, caused by overexpression of a dominant negative cadherin [99]. When *Citrobacter rodentium*-induced IBD mice were treated with the γ-secretase inhibitor Dibenzazepine (DBZ) to block Notch signaling, the AJs were affected, as demonstrated by E-cadherin and β-catenin altered expression. These mice showed signs of altered mucous makeup and bacterial dysbiosis that resulted in serious colitis and inflammation [100]. Numb is a regulatory protein that directs epithelial cell transformation to goblet cells via inhibition of Notch signaling. Co-immunoprecipitation studies conducted using Caco2 cells demonstrated that Numb interacts with E-cadherin while its downregulation compromises the epithelial barrier in a Notch signaling-independent manner [101]. Lastly, in vitro and in vivo experiments revealed Janus kinase-3 (JAK3) as a potential regulator of IBD due to its ability to control β-catenin localization at the apical junctions (Figure 2) [102].

Other conditions in the body can locally affect adherens junction integrity, leading to the development of IBD. Creatine kinases (CKs) are enzymes regulated by hypoxia-inducible transcription factors (HIFs), which fluctuate with oxygen concentrations. CKM and CKB have been shown to localize at the apical junctions, suggesting a role in regulating epithelial permeability in IBD (Figure 2); however, the exact mechanism for junction stabilization remains unclear [103]. In PIK3C3 mutant zebrafish, induction of IBD is accompanied by cytoplasmic retention and decreased localization of E-cadherin at the cell membrane of intestinal epithelial cells [104]. Although vitamin D deficiency is an unexpected candidate, it has been shown to correlate with increased risk for IBD [105] (Figure 2). An in vivo study demonstrated that vitamin D receptor null mice (*VDR^-/-^*) exhibit severe colitis [106]. In the same study, in vitro cultures of VDR-depleted Caco2 cells showed lower TEER and reduced E-cadherin levels by qRT-PCR [106]. Nevertheless, transmission electron microscope images of *VDR^-/-^* mice colons did not display significant alteration in adherens junction morphology. However, induction of 1,25-dihydroxy-vitamin D3 [1,25(OH)2 D3] increased the E-cadherin levels in SW480 colon adenocarcinoma cells [106]. Notably, genome-wide data analysis suggests an association among UC, CD, and polymorphisms in VDR [107]. Taken together, these studies demonstrate that E-cadherin junctions are a central node in a variety of mechanisms that promote barrier function and IBD progression. However, what is still missing is whether these observations can provide mechanistic insights into the reasons for the increased CRC risk for IBD patients, which remains an unresolved conundrum. Given the extensive signaling roles of E-cadherin complexes mentioned throughout this paper, this is a fertile ground for future investigation.

## 4. E-cadherin Interacts with the Colon Microbiome

**Figure 3:**
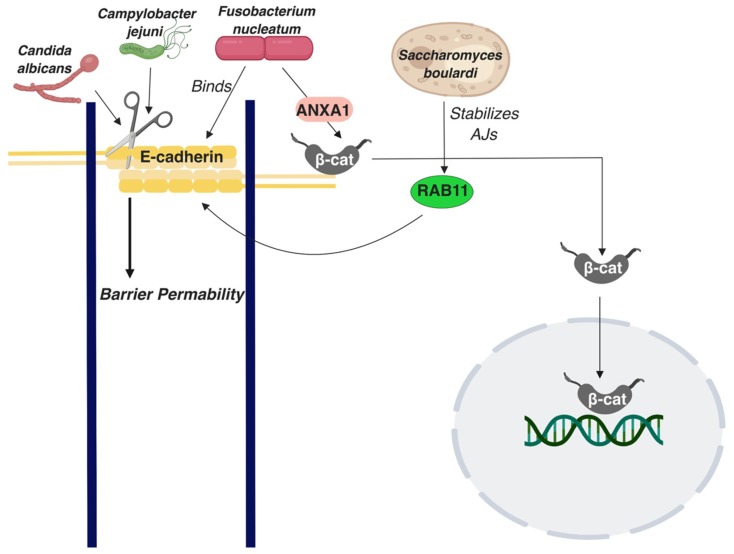
A schematic summarizing interactions of E-cadherin with the colon microbiome. Straight arrows represent activation of molecules or processes β-cat: β-catenin; p120: p120 catenin.

Projects such as Human Microbiome Project have extended our understanding of the gut microbiome, which consists of trillions of microbes. Although the commensal microbe community positively affects the overall health of the host, disturbances in the healthy microbiome, known as dysbiosis, have been shown to corelate with colon cancer occurrence [108]. The colonic epithelium acts as a barrier and blocks microorganisms from passing through. When microorganisms penetrate the epithelial barrier and enter into the inner layers, this can cause inflammation. *Bacteroides fragilis* is one such microorganism that has a positive correlation with IBD patients, both in CD and UC [109]. This bacterial species produces a metalloprotease known as Bacteroides fragilis toxin, which stimulates γ-secretase to cleave E-cadherin, resulting in AJs disruption and nuclear localization of β-catenin, ultimately promoting cell proliferation in HT29/C1 cells [110,111,112]. A study has shown that CRC patients with tumors with bacterial biofilms, which are dense bacterial populations encased in a polymeric matrix, also exhibited biofilms in their normal colonic tissue, which resulted in decreased E-cadherin expression, increased cell proliferation, and IL-6/STAT3 activation [113]. Changes in the colonic microbiome, e.g., in CRC, can affect colonic tissue homeostasis and the E-cadherin status in distant places in the colon. Notably, no specific bacterial species, but the overall presence or absence of biofilms, was associated with this phenotype. *Candida albicans* is a yeast species that has been shown to disturb the epithelial integrity of Caco2 colon epithelial cells by cleaving E-cadherin into an extracellular fragment and an intracellular fragment that acts as a substrate for γ-secretase (Figure 3) [114]. In Caco2 cells, E-cadherin was shown to be displaced from AJs when infected with *Escherichia coli* in vitro [115]. In contrast, an in vitro study conducted using HCT-8/E11 human colonic adenocarcinoma cells demonstrated that *Saccharomyces boulardi* strengthens AJs by improving E-cadherin transportation to the cell surface via regulation of recycling of Rab11-associated endosomes, (Figure 3) [97]. *Fusobacterium nucleatum* is a bacterium that directly binds E-cadherin through its Fusobacterium adhesin A (FadA) domain, promoting β-catenin signaling and stimulating proliferation in CRC cells, as confirmed by in vitro and in vivo studies [116]. Additional research has demonstrated that it is through Annexin 1 (ANXA1) that *Fusobacterium nucleatum* can mediate β-catenin signaling [117]. Work that investigated the effects of four different Lactobacillus strains on the adherens junctions of T84 colon adenocarcinoma cells noted differentially regulated E-cadherin and elevated phosphorylated β-catenin levels by some of the strains; it also noticed an overall improvement in barrier function by gram positive lactobacilli [118]. Another bacterial species, *Campylobacter jejuni*, proteolytically cleaves E-cadherin through proteases secreted in outer membrane vesicles (Figure 3) [119]. Although *Campylobacter jejuni* can be associated with inflammatory enteritis, its role in IBD is not clear. Overall, E-cadherin seems to be a critical node in the cross-talk between the intestinal epithelium and the microbiome, adding an important parameter to consider in E-cadherin’s broad role in colon homeostasis and disease.

## 5. E-cadherin as a Sensor of Physical Strain in the Colon

Colon tissues from CRC and IBD patients exhibit extensive fibrosis, characterized by increased deposition and reorganization of the extracellular matrix (ECM) [120]. Impaired barrier integrity and permeability are both causes and consequences of fibrosis [84,121]. Changes in the ECM promote physical cues and strain that can be transmitted throughout cells and tissues, altering their physiology. For example, different ECM components, such as collagen I, collagen IV, and laminin, generated different brush border enzyme expression of Caco2 cells, whereas collagen I promoted their proliferation [122]. Similarly, when Caco2 cells are put under increased strain, expression of brush border enzymes is altered [123]. Changes in the ECM also translate to changes in the stromal stiffness, which can affect cellular morphology and promote cancer progression [124]. It has been proposed that collagen has a role in this process as one the main components of ECM [125]. Indeed, HCT-8 colon cancer cells exhibit a more metastatic phenotype when cultured under low stiffness of 20–47 kPa; however, this phenotype was not observed under higher stiffness [126]. Interestingly, decrease in E-cadherin levels was also observed in cells cultured on less stiff substrates, which is in agreement with the higher metastatic potential of cells in these conditions [127]. Similarly, when colon samples from APC heterozygous mice for truncated amino acid loci 1638 were harvested and put under mechanical strain, an increase of nuclear β-catenin, MYC, and TWIST1 expression was observed [128]. A later study revealed that, when mechanical pressure was magnetically induced, phosphorylated β-catenin levels in the colon were elevated [129]. Indeed, it was shown that the Y654-β-catenin and D665-E-cadherin binding sites are affected by mechanical stress, which eventually stimulates the β-catenin signaling pathway in developing *Drosophila melanogaster* embryos [130]. The effects of mechanical stress and stiffness in the overall tumor development are described in detail in Broders-Bondon et al. (2018) [131].

Cells adhere to the ECM through integrins, which connect to the cytoskeleton and are mediators of extracellular signals. Integrins and the ECM have an intimate relationship with the AJs [132]. Integrins interact with Focal Adhesion Kinase (FAK) and Src; together, they regulate RhoGTPase activity and affect strong adhesion [133]. When Caco2 cells were put under cyclic strain, phosphorylation of JNK2 and c-MYC affected localization of E-cadherin and β-catenin while increasing epithelial permeability [134]. An in vitro study showed that TGF-β induced E-cadherin to mediate cellular adhesions in a FAK-dependent manner during ECM remodeling [135]. Overall, the data demonstrate that there is cross-talk among extracellular mechanical cues, stromal composition, and stiffness, with the integrity of the adherens junctions in the colon. Driven by these findings, further research is required to understand how the cells translate these mechanical cues to regulate junctional integrity and to better understand fibrosis and mechanical stress in the context of colon tumorigenesis.

## 6. Conclusions

E-cadherin has long been considered a critical homeostatic component of the colonic epithelium, primarily due to its central role in cellular architecture and barrier function. It has also been thought to primarily act as a tumor suppressor in CRC. However, numerous emerging roles of E-cadherin in intracellular signaling and cell behavior as well as its extensive cross-talk with the colonic epithelial microenvironment reveal a broader and more complicated role. Furthermore, the identification of new E-cadherin partners at the AJs add to the complexity, introducing new aspects and questions in cadherin biology. These recent findings portray E-cadherin and of the AJs as not merely structural components of cells and tissues but in new roles as signaling hubs, opening novel and exciting avenues of investigation.

## Abbreviations

AJ	Adherens Junction
CD	Crohn’s Disease
CRC	Colorectal Cancer
DISC	death-inducing signaling complex
DSS	Dextran Sulfate Sodium
ECM	extracellular matrix
EMT	Epithelial to Mesenchymal Transition
IBD	Inflammatory Bowel Disease
NE	neutrophil elastase
p120	p120 catenin
PMNs	Polymorphonuclear neutrophils
qRT-PCR	quantitative reverse transcription polymerase chain reaction
RNAi	RNA interference
RISC	RNA-induced silencing complex
TEER	trans-epithelial electrical resistance
TJ	tight junction
UC	Ulcerative Colitis
ZA	zonula adherens
